# CPAnet Registry—An International Chronic Pulmonary Aspergillosis Registry

**DOI:** 10.3390/jof6030096

**Published:** 2020-06-29

**Authors:** Christian B. Laursen, Jesper Rømhild Davidsen, Lander Van Acker, Helmut J.F. Salzer, Danila Seidel, Oliver A. Cornely, Martin Hoenigl, Ana Alastruey-Izquierdo, Christophe Hennequin, Cendrine Godet, Aleksandra Barac, Holger Flick, Oxana Munteanu, Eva Van Braeckel

**Affiliations:** 1 Pulmonary Aspergillosis Centre Denmark (PACD), Department of Respiratory Medicine, Odense University Hospital, 5000 Odense C, Denmark; Christian.B.Laursen@rsyd.dk (C.B.L.); jesper.roemhild.davidsen@rsyd.dk (J.R.D.); 2 Institute of Clinical Research, University of Southern Denmark, 5000 Odense C, Denmark; 3 Department of Respiratory Medicine, Ghent University Hospital, B-9000 Ghent, Belgium; Lander.VanAcker@UGent.be; 4 Department of Internal Medicine and Paediatrics, Ghent University, B-9000 Ghent, Belgium; 5 Department of Pulmonology, Kepler University Hospital, 4021 Linz, Austria; helmut.salzer@kepleruniklinikum.at; 6 Department of Internal Medicine, Faculty of Medicine and University of Cologne, Excellence Center for Medical Mycology (ECMM), University of Cologne, 50923 Cologne, Germany; danila.seidel@uk-koeln.de (D.S.); oliver.cornely@uk-koeln.de (O.A.C.); 7 Cologne Excellence Cluster on Cellular Stress Responses in Aging-Associated Diseases (CECAD), University of Cologne, 50923 Cologne, Germany; 8 German Centre for Infection Research (DZIF), Partner Site Bonn-Cologne, 50923 Cologne, Germany; 9 Division of Infectious Diseases and Global Health, University of California San Diego, La Jolla, CA 92037, USA; mhoenigl@health.ucsd.edu; 10 Section of Infectious Diseases and Tropical Medicine, Medical University of Graz, 8036 Graz, Austria; 11 Mycology Reference Laboratory, National Centre for Microbiology, Instituto de Salud Carlos III, 28220 Madrid, Spain; anaalastruey@isciii.es; 12 Sorbonne Université, Inserm, Centre de Recherche Saint-Antoine, CRSA, AP-HP, Hôpital Saint-Antoine, Service de Parasitologie-Mycologie, F-75012 Paris, France; christophe.hennequin-sat@aphp.fr; 13 Department of Pulmonology, AP-HP, Hôpital Bichat, 75018 Paris, France; cendrine.godet@aphp.fr; 14 Clinic for Infectious and Tropical Diseases, Clinical Centre of Serbia, 11000 Belgrade, Serbia; aleksandrabarac85@gmail.com; 15 Faculty of Medicine, University of Belgrade, 11000 Belgrade, Serbia; 16 Division of Pulmonology, Department of Internal Medicine, Medical University of Graz, 8036 Graz, Austria; Holger.Flick@klinikum-graz.at; 17 Division of Pneumology and Allergology, Department of Internal Medicine, State University of Medicine and Pharmacy “Nicolae Testemitanu”, MD-2004 Chisinau, Republic of Moldova; oxana.munteanu@usmf.md

**Keywords:** *Aspergillus*, chronic pulmonary aspergillosis, diagnosis, treatment, antifungals, registry, international collaboration, CPAnet

## Abstract

Chronic pulmonary aspergillosis (CPA) is a chronic fungal infection of the lung associated with high morbidity and mortality. The CPA Research network (CPAnet) registry established in 2018 is an international multicenter collaboration aiming to improve CPA knowledge and patient care. This study’s aim was to describe the data collection process and content of CPAnet registry with preliminary clinical data. In the CPAnet registry, clinical data are collected through a web-based questionnaire. Data include CPA phenotype, comorbidities, treatment, outcome, and follow-up from several international centers. An exemplary descriptive analysis was performed on 74 patients, who were registered online before April 2020. CPA patients were predominantly (72%) male, 39% had chronic obstructive pulmonary disease, and 68% had a history of smoking. Chronic cavitary pulmonary aspergillosis was the most common CPA subtype (62%). In 32 patients (52%), voriconazole was the preferred first-line therapy. The multicenter multinational CPAnet registry is a valuable approach to gather comprehensive data on a large study population and reflects real-world clinical practice rather than focusing on specific patient populations in more specialized centers. Additional CPA reference centers are being encouraged to join this promising clinical research collaboration.

## 1. Introduction

Chronic pulmonary aspergillosis (CPA) is a disease spectrum consisting of different phenotypes of severe chronic fungal infection of the lung [1,2]. CPA patients are hampered by a substantial morbidity affecting approximately 3 million people worldwide and with an overall 5-year mortality of up to 80% that estimates 450,000 annual deaths [3,4,5]. Despite this apparently high disease burden, paradoxically CPA prevalence seems to be low in single centers [6]. There might be several explanations for this appearance. The clinical and radiological presentation of CPA is rarely obvious since CPA most often occurs as a continuum of overlapping syndromes, in which one CPA phenotype can transform into another [7,8]. Likewise, CPA diagnosis can easily be overlooked as it often occurs with insidious symptoms superimposed on the patient’s pre-existing pulmonary disease. Chronic obstructive pulmonary disease (COPD), prior tuberculosis (TB), non-tuberculous mycobacterial (NTM) infection, sarcoidosis, prior pneumothorax, and allergic bronchopulmonary aspergillosis (ABPA) comprise over 90% of the other underlying conditions [9,10,11].

Physicians’ unawareness or negligence of CPA in a patient is an important risk factor for diagnostic and therapeutic delay, which partly explains the increased mortality and the observed low single-site prevalence. Since many aspects of this underestimated disease are unknown, a prerequisite for improving the lack of knowledge is to join experience and expand data gathering across CPA reference centers worldwide. On this basis, the international CPA Research Network (CPAnet) was founded in 2017, followed by the creation of its multinational and multicenter CPAnet registry launched in 2018 [12,13]. The main objective of the CPAnet registry is to assess the worldwide epidemiology of CPA, while the secondary objectives include the evaluation of diagnostic performance and treatment outcome measures across the different participating centers. Eventually, this registry will enable longitudinal studies monitoring the disease burden and clinically oriented research in order to improve the management and outcome of CPA patients [14].

The aim of this article is to describe the CPAnet registry and present preliminary multicentric data on CPA patients to illustrate the clinical value and importance of such an international registry.

## 2. Materials and Methods

The CPAnet registry is an open international registry containing individual and pseudonymized data from CPA patients from the participating reference centers, eventually giving rise to epidemiological and clinical non-interventional multicenter studies. CPAnet registry was officially launched in 2018 and data collection is ongoing without an endpoint. As the registry is open, treating physicians from various specialties (e.g., pulmonology or infectious diseases) and microbiologists are invited to participate in the registry to further expand the extent of clinical data collection.

### 2.1. CPA Case Documentation, Definitions, and Data Collection

CPAnet developed a case report form using the ClinicalSurveys.net online survey platform (QuestBack GmbH, Cologne, Germany). Investigators log into the system with username and password and are able to view and modify their own contributions. All data transmissions are encrypted via transport layer security (TLS) 1.2 with an advanced encryption standard (AES) 256 Galois/Counter mode (GCM) bit key and ephemeral elliptic curve Diffie-Hellman (ECDHE) Rivest-Shamir-Adleman (RSA) key exchange. Data are documented pseudonymously or anonymously on QuestBack servers.

Adult patients with a diagnosis of CPA are included either prospectively or retrospectively. The population is selected based on the following four diagnostic criteria [1,15]: (1) Radiology: one or more cavities with or without a fungal ball present or nodules on thoracic imaging; (2) Mycology: any direct or indirect mycological evidence from respiratory samples or from blood of *Aspergillus* spp. infection; (3) Differential diagnosis: exclusion of an alternative diagnosis (e.g., lung cancer); (4) Chronic disease: disease present for at least 3 months (e.g., chronic respiratory symptoms ≥3 months or available follow-up images with CPA features ≥3 months).

Upon entry in the registry, the CPA phenotype is based on the criteria and definitions previously described by Denning et al. [1]: *Aspergillus* nodule(s): defined as one or more nodules, which may or may not cavitate; can only be definitively diagnosed on histology; necrosis may be present, but tissue invasion is not demonstrated. Single/simple aspergilloma: single pulmonary cavity containing a fungus ball. Chronic cavitary pulmonary aspergillosis (CCPA): one or more pulmonary cavities (with either a thin or thick wall) possibly containing one or more aspergillomas or irregular intraluminal material. Chronic fibrosing pulmonary aspergillosis (CFPA): severe fibrotic destruction of at least two lung lobes complicating untreated CCPA; usually the fibrosis is manifest as consolidation, but large cavities with surrounding fibrosis may be seen. Subacute invasive aspergillosis (SAIA): radiologically similar to CCPA, but a more rapidly progression (< 3 months), and usually found in moderately immunocompromised patients; histology shows hyphae with tissue invasion and microbiological investigations reflect those of invasive aspergillosis.

After inclusion in the registry, the following baseline data are gathered concerning the primary endpoint of collecting epidemiological data: CPA phenotype, time point of diagnosis, age at diagnosis, sex, familial disposition, CPA risk factors (e.g., pulmonary disorders, previous invasive pulmonary aspergillosis (IPA), pharmacological and/or constitutional immunosuppression, behavioral factors (e.g., smoking, abuse of drugs or alcohol), other chronic diseases (e.g., diabetes; heart-, liver-, and kidney disease), pulmonary co-infection at CPA diagnosis time point. Regarding the secondary endpoint of CPA diagnostic performance, clinical signs and symptoms, weight, height, lung function test(s), result of clinical scores (e.g., St. George’s Respiratory Questionnaire (SGRQ), Medical Research Council dyspnea scale (MRC)), result of imaging and mycological testing leading to CPA diagnosis (e.g., microscopy, culture, polymerase chain reaction (PCR), histology, *Aspergillus* precipitins, *Aspergillus*-specific IgG antibody, galactomannan assay, beta-D-glucan assay), result of antifungal susceptibility testing are being collected. As treatment outcome measures, the following data are requested: antifungal treatment of CPA (e.g., drug, length, response, reason for treatment being stopped), use of therapeutic drug monitoring, and other CPA treatment measures (e.g., surgery, embolization). At later timepoints, additional information on survival and status of CPA disease is collected corresponding to follow-up at 6 months, 2 years, and 10 years following CPA diagnosis.

### 2.2. CPA Case Recruitment

Eligible CPA cases are identified at sites participating in the CPAnet registry, mostly clinical departments or centers responsible for the diagnosis, treatment, and follow-up of CPA in their respective regions or countries (Table 1). Additional reference centers are being welcomed to join the CPAnet registry. At each center, cases are identified using relevant diagnosis codes (e.g., ICD-10) for *Aspergillus*-related disease, local CPA registries, and medical records. Incident CPA cases fulfilling the above-mentioned diagnostic criteria are included both prospectively and retrospectively in the registry. The investigators at each site including patients and entering data are all specialists within the field of CPA. Diagnostic tools, treatment, and monitoring at each site are applied in accordance with current international guidelines and recommendations.

### 2.3. Quality Control

Following the entry of a case in the registry, a clinical data manager and respiratory infectious diseases specialist review the case for completeness and consistency. Subsequently, queries are issued in case of missing or inconclusive data. Formal queries are only sent to the specific site in the case of substantial missing or unclear information impeding further evaluation with regard to the medical content. Changes will be made by the contributing party for each case accordingly. After resolution of queries, cases are considered valid and available for analysis.

### 2.4. Ethical Considerations and Approvals

The CPAnet registry is approved by the Institutional Review Board and Ethics Committee (EC) of the University Hospital Cologne (procedure ID 17-263), Cologne, Germany, as well as by the local ECs of the other participating centers. For prospectively included cases, oral and written informed consent from the patients has to be provided in order to participate. Approval for inclusion of retrospective cases has been obtained locally at each site in accordance with the different local requirements. All patient data are pseudonymized and stored in accordance with the General Data Protection Regulation (GDPR).

### 2.5. Funding

At the time of the writing of this manuscript, the CPAnet registry has not received any financial support. Support for the initial programming and maintenance of the database was provided by Excellence Center of Medical Mycology (ECMM), Hospital Cologne, Cologne, Germany. Up to date, sites participating in CPAnet do not receive any financial support or compensation for their participation in the registry. As of February 2020, the CPAnet registry was awarded a Clinical Research Collaboration (CRC) by the European Respiratory Society (ERS) for the next three years.

### 2.6. Statistical Analysis

Descriptive statistics were performed by use of SPSS Statistics software version 26.0. Statistical testing of categorical variables was performed making use of chi-square tests with statistical significance determined at *p* < 0.05.

## 3. Results

A first and exemplary interim analysis was performed on 74 patients, retrospectively and prospectively included from 2018 up to April 2020. The patients were recruited from eight centers and six countries, all within Europe (Table 1).

### 3.1. Demographics

In the current cohort, the mean age was 57 years, with a male predominance (72%). CCPA was the most frequently diagnosed phenotype (*n* = 46 (62%)). All patients had predisposing pulmonary risk factors. Sixty-eight percent had a history of (current) smoking. COPD (39%) and severe asthma (26%) were the most frequent underlying lung diseases. Only 20% of patients had active or previous pulmonary tuberculosis. Many patients additionally had non-pulmonary comorbidities, such as diabetes (11%) or cardiovascular disease (19%). At least 35% of patients were known to have some degree of immunosuppression, mainly induced by systemic corticosteroid use (19 patients (26%)) or underlying diseases associated with immunosuppression (12 patients (19%)) (Table 2).

Co-infections with other respiratory pathogens were frequent (22%), mainly with *Pseudomonas aeruginosa* (*n* = 4) or *Mycobacterium tuberculosis* (*n* = 3). Only two patients were co-infected with non-tuberculous mycobacteria (NTM), respectively with *Mycobacterium kansasii* and *Mycobacterium xenopi*, and two patients were reported to be co-infected with Mucorales.

### 3.2. Diagnosis of CPA

From a clinical point of view, patients often presented with productive cough (88%) and dyspnea (76%). Mild or more severe hemoptysis was mentioned in 35 cases (47%). Weight loss (51%) and fatigue (55%) were the most prevalent general complaints.

CCPA with an intracavitary fungus ball was the most common radiological feature (68%), with surrounding *Aspergillus* nodules, pleural disease, bronchiectasis, and consolidations also frequently being detected. Rather rarely observed radiological signs were air trapping, bronchiolitis, hydropneumothorax, pleural effusion, pulmonary embolism, bullae, and bronchopleural fistula. Overall, the upper lobes were most frequently affected, in 56 patients (76%) the right upper lobe and in 31 patients (42%) the left upper lobes.

Microbiological diagnosis was mostly based on serum *Aspergillus*-specific IgG (elevated in 48 of 62 patients) and *Aspergillus* antigen (galactomannan) on bronchoalveolar lavage (BAL) (positive in 28 of 56 patients). In 20% of patients, molecular detection of *Aspergillus* (AsperGenius^®^) was performed on various specimen (sputum, BAL, biopsy). A positive sputum and BAL culture were present in, respectively, 28% and 22% of patients. In 53% of patients, no positive culture was documented (not available or negative). *Aspergillus fumigatus* was the most commonly identified species (*n* = 49), detected directly by culture or indirectly through antibody response (66%), followed by *Aspergillus niger* (*n* = 11) (Table 3).

### 3.3. Treatment and Outcome

Overall, voriconazole was most frequently administered as a first line (52%), followed by itraconazole (42%). Both were given in 48% of CCPA patients as first-line treatment. In SAIA, voriconazole was the preferred antifungal (52%). Clinical and radiological improvement were documented in, respectively, 53% and 38% of CPA patients. Treatment failure in first line was rare (5%); nevertheless, treatment duration was highly variable among patients (0–31 months) (Table 4).

The clinical and radiological response was significantly higher for voriconazole, in 22 of 27 and 19 of 26 patients (*p* = 0.020), respectively, than that for itraconazole, in 8 of 24 and 3 of 17 patients (*p* = 0.010, respectively). A positive *Aspergillus* IgG or BAL galactomannan was present in 90% or 66% of patients treated with voriconazole in first line, respectively. For those treated with itraconazole, these numbers were lower (65% and 45%, respectively) (Table 5).

Reasons for stopping therapy after first-line treatment were mainly treatment completion (31%) or drug-related adverse events (28%). Forty-two percent of patients received second-line therapy. Surgery was performed in 13.5% of patients (Table 4).

The mean first-line treatment duration was 5.6 months (range 0–31). Although 47% mentioned hemoptysis at diagnosis, in only 4% of the patients, a bronchial artery embolization was performed during follow-up. Five percent received surgery for several reasons (e.g., pneumothorax). Three patients died during follow-up due to concomitant pneumococcal septic shock with acute respiratory distress syndrome (ARDS), post-procedural aspiration-induced ARDS, and a myocardial infarction, respectively (Table 4).

## 4. Discussion

This first interim analysis of the multicentric multinational CPAnet registry revealed a very similar study population to earlier European studies [14,16,17]. It contained mainly older males with a smoking history. Dyspnea and chronic cough were the most frequent symptoms and hemoptysis occurred in almost half of the cases. CCPA was the most common form of CPA in this registry, but throughout the history of several cases, the difficult characterization and overlap between the different CPA subtypes became clear. Sometimes these subtypes transformed during follow-up into another subtype as well, further illustrating the fact that CPA is a disease spectrum [1,2].

COPD was the predominant comorbidity, in line with existing literature (29% to 42% of CPA patients). The prevalence of a history of *M. tuberculosis* infection (20%) was similar to other European studies of Salzer et al. (24%) [16] and Bongomin et al. (18%) [17], but lower than in the cohort of Godet et al. (38.9%) [14] and in population studies worldwide (30–81%). Of note, the majority of patients included in the registry until today, originated from tertiary care centers in low tuberculosis incidence regions. Surprisingly, the increasing incidence of NTM co-infections could not be confirmed in this cohort (only 3% versus 10–11% in earlier reports) [14,16,17].

One third of patients in our registry were considered immunosuppressed to some extent, mainly by oral corticosteroid use or underlying auto-immune disease. Immunosuppression is a controversial item in CPA, and the exact role and mechanism of immunologic defects in the pathogenesis are still unclear. Therefore, the Infectious Diseases Society of America (IDSA) guidelines excluded immunosuppression from their diagnostic criteria, while the ERS guidelines state that only in the case of SAIA, mild immunodeficiency is common [1,18]. As a consequence, most studies do not describe immunosuppression as an important comorbidity. Only 11.3% of German patients in previous reports were immunocompromised, and 8% had an auto-immune disease [16]. However, in a French cohort, 46% of patients were on oral steroids, which is even higher than reported here (26%) [14].

In analogy with the ERS and IDSA guidelines, the most contributable microbiological evidence in this cohort was primarily serum *Aspergillus* IgG and secondly *Aspergillus* antigen detected in a respiratory specimen [1,18]. *A. fumigatus* (66%)*,* followed by *A. niger* (15%), was the most frequent culprit species. Sputum and BAL culture contributed only in 28% and 22% of cases to the diagnosis, which confirms the rather low sensitivity of culture (for 53% culture was either not available or negative). Transbronchial biopsy for *Aspergillus* PCR, culture, and/or histology was a rather unexpected, useful diagnostic tool in several patients (12%). Despite its invasive character, surgery remains very effective in diagnosing CPA (positive results in 9 out of 10 patients).

Voriconazole was the preferred first-line treatment (52%), followed by itraconazole (42%). This is congruent with other studies and the current guidelines, where both are recommended as first-line treatments [3,17,19]. Patient characteristics, such as CPA subtype and disease burden, might play a role in the treatment choice. Voriconazole was the preferred treatment in patients with SAIA (52% vs. 40% on itraconazole), which is in line with the European guidelines [1]. A positive *Aspergillus* IgG was far more frequent in the group with voriconazole (90%) than in the one with itraconazole (65%). One might speculate on a preference for the use of voriconazole in case of a higher disease burden. Although a similar observation was seen in the study of Bongomin, guidelines do not rely on *Aspergillus* IgG for disease severity [1,17]. Moreover, these results can be biased, because in simple aspergilloma, which is more often treated with itraconazole, *Aspergillus* IgG is generally less detected. The possible influence of local treatment guidelines and reimbursement issues among the different countries has to be taken in account as well.

All patients treated with voriconazole as first line experienced a high rate of clinical (81%) and radiological (73%) improvement. These rates are higher than those in other studies, but this could be biased by the unstandardized follow-up time and the fact that many patients needed to switch to another drug because of an adverse event (28%). On the other hand, the clinical response rate to itraconazole was significantly lower than expected. A similar observation was seen in a recent retrospective observational study in Japan, where voriconazole showed better effectiveness than itraconazole for clinical improvement, but this effect was erased when stable patients were included [20]. In our cohort, we also noted a high percentage of stable disease in patients with itraconazole. This can partially be explained by the tendency to start itraconazole for treatment of simple aspergilloma or in milder disease, when patients are often less symptomatic and radiological response takes some time. It confirms the recommendation that a watch-and-wait approach can be a good alternative for simple aspergilloma, because this usually does not respond well to medical treatment [18,19].

There were too little data to evaluate the role of posaconazole and isavuconazole. Posaconazole can be a good alternative in patients who do not respond sufficiently to voriconazole. Isavuconazole’s favorable safety profile will probably make it an attractive alternative for voriconazole in the future, although there are no robust data yet in the long-term treatment of pulmonary aspergillosis.

The main strength of the CPAnet registry is that it collects real-life data on an international level, which renders overall quality assessment of CPA management and also reveals tendencies on selected aspects requiring improvement, e.g., clarification of causes to diagnostic latency. Another strength is that the main proportion of CPA data is based on multidisciplinary team discussions involving experts of different backgrounds who follow international consensus on CPA management. By linking data from other registries with the CPAnet registry, specific research questions can be uncovered, such as pharmaco-epidemiological studies on medication patterns giving rise to or increasing the risk of CPA development, e.g., combination of certain immunosuppressants in chronic underlying diseases.

The major limitation is that not all CPA patients are entered in the CPAnet registry, which makes the data subject to selection bias. As such, one may claim that data are not generalizable but only concern patients from tertiary care centers, thereby inducing referral bias. Another important limitation is the different follow-up times of the enrolled patients and the non-standardized (timing of) response evaluation. Of note, the registry currently integrates retrospective and prospective data as some patients were already under follow-up at the time of informed consent or only referred to the CPA reference center after failure of first-line treatment. As the project progresses, however, a shift toward more prospective follow up might be anticipated. Data quality might vary between centers due to different reasons, such as infrastructural or interpersonal differences. Although patient numbers remain too low to draw any firm conclusions, this first analysis already revealed the unavailability of some diagnostic techniques (such as galactomannan) or therapeutic options (such as certain antifungals or the availability of interventional radiology for bronchial artery embolization) in certain regions. This registry might help in identifying and advocating for regions in need of more resources or access to state-of-the-art diagnostics and therapeutics.

## 5. Conclusions

The CPAnet registry is the first international registry of CPA to our knowledge, increasing awareness among pulmonologists and infectious diseases specialists worldwide and allowing for future clinical and epidemiological research. As the registry is not restricted to certain centers or countries, there is a great potential of exploring, amongst others, the incidence and prevalence over time, referral patterns for CPA centers, risk factors of CPA development, but also predictors for disease severity and mortality. Altogether, this information might guide clinicians toward improved disease management, by means of the right targeted antifungal treatment and appropriate treatment duration, and standardization of follow-up and response evaluation. International CPA tendencies based on observations and results from the CPAnet registry may eventually contribute to improvement in diagnostic and therapeutic decision making and indirectly have impact on future guidelines. Data on referral patterns may also reveal bottlenecks for the apparent diagnostic delay or neglect that seems to exist in low-prevalence countries where the prevalence of comorbidities on the other hand is similar to that of countries with a higher CPA prevalence.

This interim analysis lifts a first tip from the veil of the wealth of structured information embedded within the CPAnet registry. Additional clinical sites and reference centers are highly welcomed to join this clinical research collaboration. In the near future, this registry will indefinitely allow for multicentric clinical and epidemiological research on CPA of crucial importance, aiming to fill the lack of previous evidence on disease management.

## Figures and Tables

**Table 1:** Sites currently recruiting patients for the Chronic Pulmonary Aspergillosis Research Network (CPAnet) registry.

Country	Institution	Recruitment Status: n (%)
Belgium	Ghent University Hospital	27 (36%)
Denmark	Odense University Hospital	21 (28%)
Moldova	State University of Medicine and Pharmacy, Chisinau	12 (16%)
Serbia	University of Belgrade	5 (7%)
Germany	University of Cologne	3 (4%)
	Asklepios Pulmonary Clinic, Munich-Gauting	3 (4%)
	Evangelical Lung Clinic, Berlin	1 (1%)
United Kingdom	University of Manchester	2 (3%)
Austria	Kepler University Hospital, Linz	Open for inclusion

**Table 2:** Demographics of patients included in CPAnet.

Patient Characteristic	Number (Total *n* = 74)	Percentage (%)
**CPA phenotype**		
Simple aspergilloma	8	11
CCPA	46	62
CFPA	3	4
SAIA	14	19
*Aspergillus* nodule	3	4
**Demographics**		
Age in years (range)	57 (16–88)	
Male	53	72
Female	21	28
BMI (range)	22 (13–40)	
**Pulmonary risk factors**	71	96
Previous/active smoking	50	68
COPD	29	39
Asthma	19	26
Co-infection	16	22
History of tuberculosis	12	16
Thoracic surgery	10	14
Idiopathic bronchiectasis	9	12
ABPA	8	11
Thoracic radiotherapy	6	8
Pneumothorax	3	4
History of IPA	3	4
Sarcoidosis	3	4
Active tuberculosis	3	4
Active/history of NTM-PD	2	3
Lung cancer	7	10
Familial history	0	0
**Non-pulmonary risk factors**		
Lifestyle (alcohol/drug abuse, obesity/underweight)	16	22
Low BMI (<18)	14	19
Cardiovascular disease	14	19
Alcoholism	13	18
Rheumatological disease	9	12
Diabetes	8	11
Obesity	4	5
Liver disease	3	4
Renal disease	2	3
**Immunosuppression**	26	35
Oral corticosteroids	19	26
Immunosuppressive drug other than steroids	8	11
Allogeneic stem cell transplant	3	4
HIV infection	1	1
Other disease associated with immunosuppression	12	16
GPA	2	3
RA	4	5
Sarcoidosis	2	3
Psoriasis	1	1
Melanoma	1	1
HIES	1	1
PID	1	1

ABPA: allergic bronchopulmonary aspergillosis; BMI: body mass index; CCPA: chronic cavitary pulmonary aspergillosis; CFPA: chronic fibrotic pulmonary aspergillosis; COPD: chronic obstructive pulmonary disease; CPA: chronic pulmonary aspergillosis; GPA: granulomatosis with polyangiitis; HIES: hyperimmunoglobulin E syndrome; HIV: human immunodeficiency virus; IPA: invasive pulmonary aspergillosis; NTM-PD: non-tuberculous mycobacteria pulmonary disease; PID: primary immunodeficiency; RA: rheumatoid arthritis; SAIA: subacute invasive aspergillosis. In bold: subheaders

**Table 3:** Diagnosis of chronic pulmonary aspergillosis (CPA).

Parameter	Number (Total *n* = 74)	Percentage (%)
**Presenting symptoms**	72	96
Cough	65	88
Sputum	57	77
Dyspnea	56	76
Fatigue	41	55
Weight loss	38	51
Hemoptysis	35	47
Chest pain	25	34
**Radiological fungal signs**		
Intracavitary *Aspergillus*	50	68
Cavity without fungus	25	34
*Aspergillus* nodules	22	30
Single *Aspergillus* nodule	7	10
Aspergilloma	6	8
**Other radiological signs**		
Bronchiectasis	49	66
Pleural disease	41	55
Consolidation	34	46
Emphysema	33	45
Lymphadenopathy	26	35
Fibrosis	17	23
Pneumothorax	2	3
**Microbiology**		
*Aspergillus* IgG	48 (of 62)	65 (77% of tested)
*Aspergillus* lateral flow assay	4	5
Positive culture (ever)	35	47
–Sputum culture	21	28
–BAL culture	16	22
BAL/sputum/TBB PCR	15	20
BAL galactomannan		
–at diagnosis	24 (of 40)	32 (60% of tested)
–ever documented	28 (of 56)	38 (50% of tested)
Serum galactomannan	4	5
Beta-d glucan	4	5
***Aspergillus* species**		
No type (no IgG or culture)	18	24
*Aspergillus fumigatus*	49	66
*Aspergillus niger*	11	15
*Aspergillus flavus*	2	3
**Diagnosis after biopsy**		
Transthoracic puncture *	6	8
Transbronchial biopsy *	9	12
Surgical biopsy *	9 (9/10)	12 (90%)

BAL: bronchoalveolar lavage; IgG: immunoglobulin G; PCR: polymerase chain reaction; TBB: transbronchial biopsy. * Histology or microbiology. In bold: subheaders

**Table 4:** Treatment and outcome of CPA patients.

Treatment Choice	Number (Total *n* = 74)	Percentage (%)
**First-line treatment**	62	Percentage of subtotal
Voriconazole	32	52
Itraconazole	26	42
Posaconazole	3	5
Amphotericin B	1	2
**First-line treatment CPA type**		
CCPA	42	Percentage of subtotal
–Voriconazole	20	48
–Itraconazole	20	48
–Posaconazole	2	5
SAIA	23	Percentage of subtotal
–Voriconazole	12	52
–Itraconazole	9	40
–Posaconazole	1	4
–Amphotericin B	1	4
Aspergilloma	6	Percentage of subtotal
–Voriconazole	2	33
–Itraconazole	4	66
*Aspergillus* nodule	2	Percentage of subtotal
–Voriconazole	1	50
–Itraconazole	1	50
**Reason for therapy cessation**	58	Percentage of subtotal
No stop	15	26
Treatment completion	18	31
Treatment switch	6	10
Drug-related adverse event	16	28
Failure	3	5
**Clinical response first-line**	60	Percentage of subtotal
Improvement	32	53
Stable	17	28
Progression	5	8
**Radiologic response first-line**	60	Percentage of subtotal
Improvement	23	38
Stable	19	31
Progression	3	5
**Second-line treatment**	31	Percentage of subtotal
Voriconazole	12	39
Itraconazole	7	23
Posaconazole	8	26
Amphotericin B	2	7
Caspofungine	1	3
Amphotericin B + Voriconazole	1	3
**Other treatment**		
Surgery for treatment	10	13.5
Surgery for complications	4	5
Embolization	4	5
**Death**	3	4

CCPA: chronic cavitary pulmonary aspergillosis; CPA: chronic pulmonary aspergillosis; SAIA: subacute invasive aspergillosis. In bold: subheaders

**Table 5:** Diagnostic and outcome parameters in CPA patients treated with voriconazole and itraconazole. In bold: subheaders.

Outcome	Voriconazole		Itraconazole		*p*-Value
	Number (Total *n* = 32)	Percentage (Subtotal)	Number (Total *n* = 26)	Percentage (Subtotal)	
**First-line clinical response**	27 (5 missing)		24 (2 missing)		0.020
Improvement	22	81	8	33	
Stable	5	19	11	46	
Progression	0	0	5	21	
**First-line radiological response**	26 (6 missing)		17 (9 missing)		0.010
Improvement	19	73	3	18	
Stable	7	27	11	65	
Progression	0	0	3	18	
***Aspergillus* IgG positivity**	26 (3 missing)	90	15 (3 missing)	65	0.016
***Aspergillus* antigen positivity**	16 (8 missing)	66	10 (4 missing)	45	0.244
**Treatment cessation**	32		24 (2 missing)		
Failure	1	3	2	8	
Drug-related adverse event	9	28	6	25	
